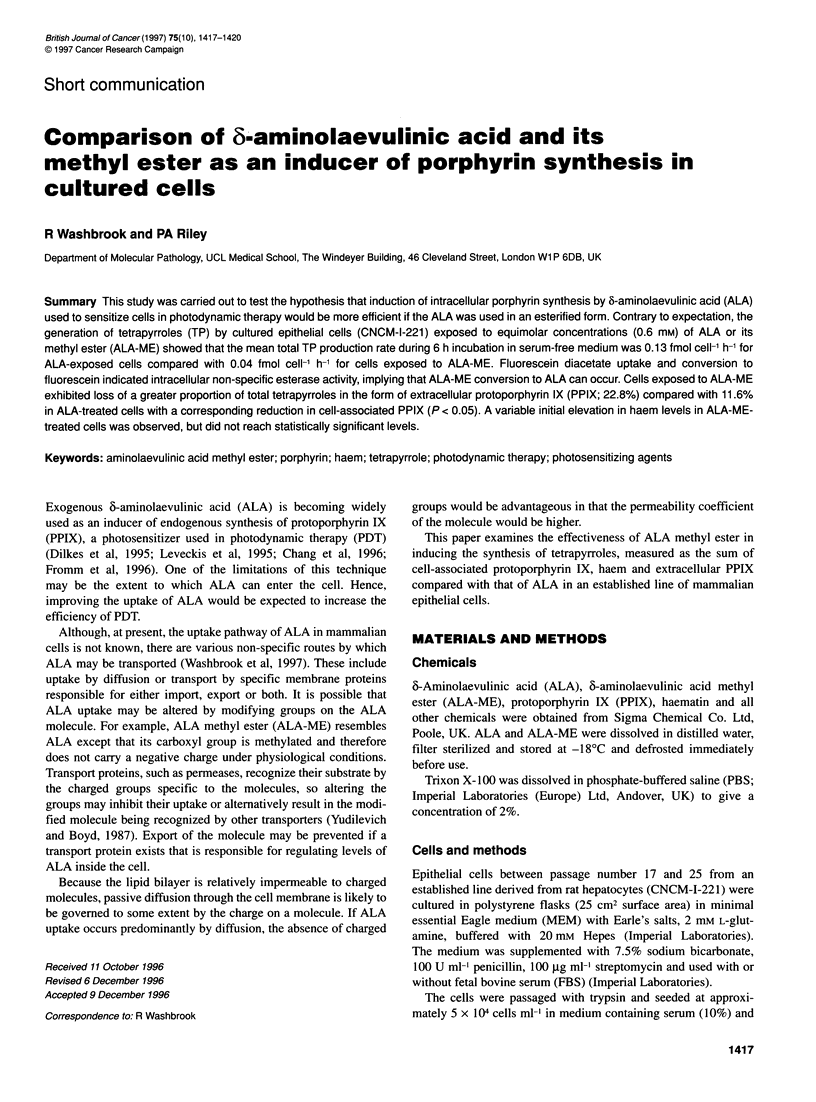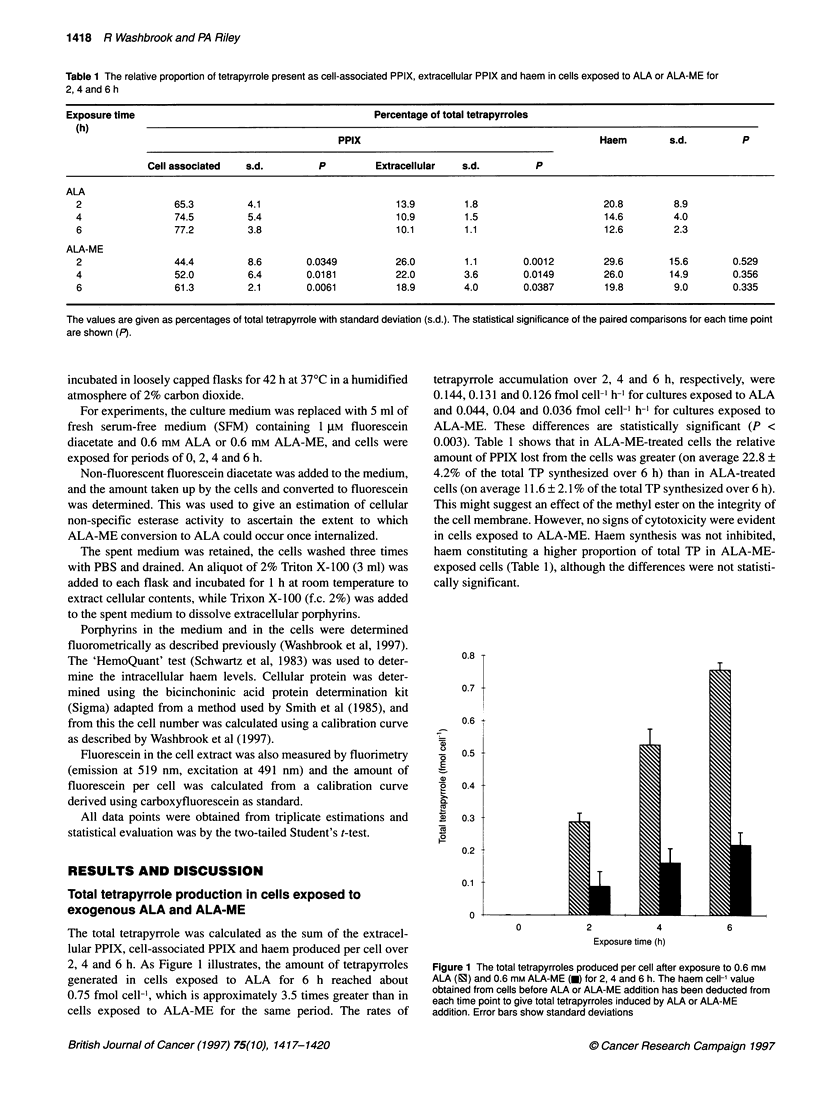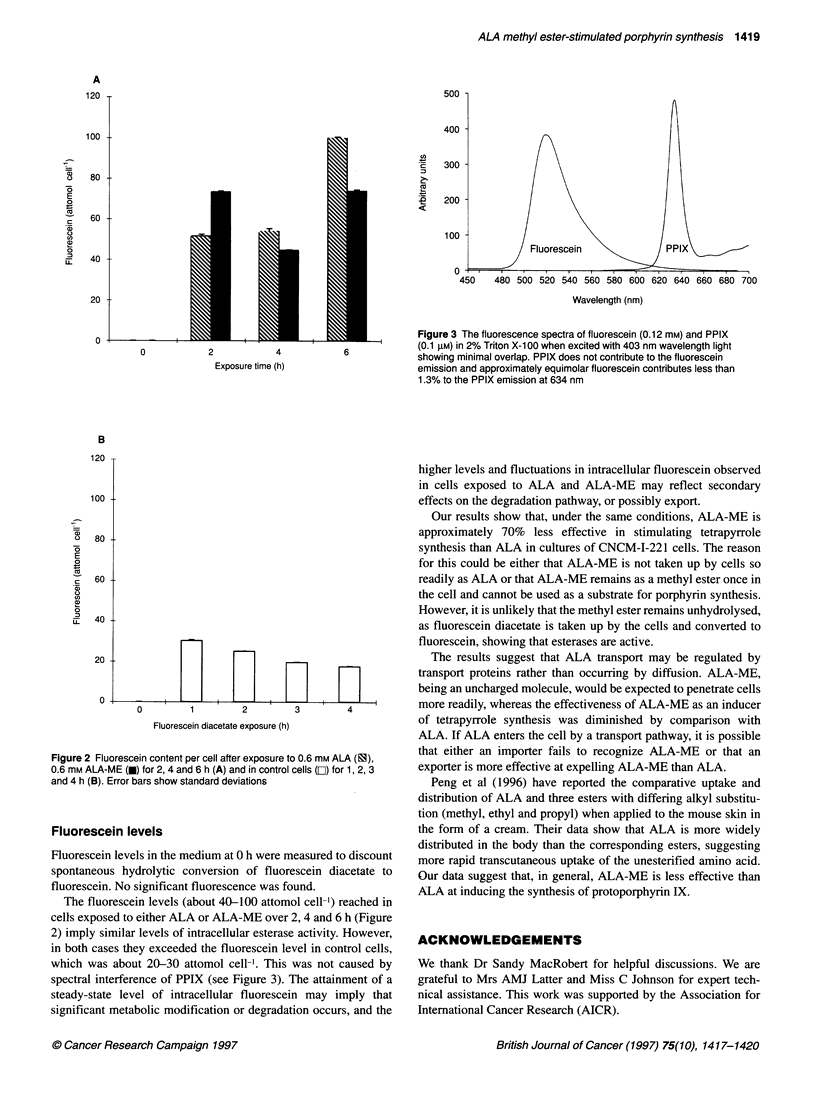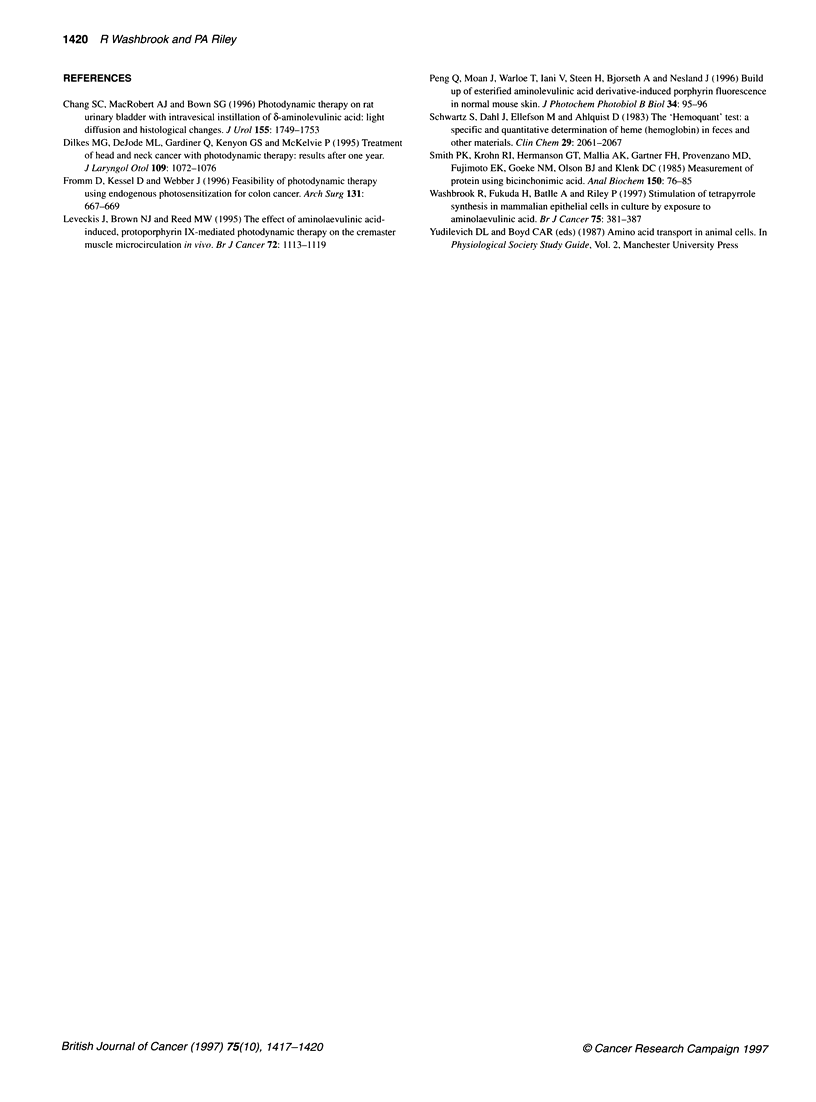# Comparison of delta-aminolaevulinic acid and its methyl ester as an inducer of porphyrin synthesis in cultured cells.

**DOI:** 10.1038/bjc.1997.244

**Published:** 1997

**Authors:** R. Washbrook, P. A. Riley

**Affiliations:** Department of Molecular Pathology, UCL Medical School, London, UK.

## Abstract

This study was carried out to test the hypothesis that induction of intracellular porphyrin synthesis by delta-aminolaevulinic acid (ALA) used to sensitize cells in photodynamic therapy would be more efficient if the ALA was used in an esterified form. Contrary to expectation, the generation of tetrapyrroles (TP) by cultured epithelial cells (CNCM-I-221) exposed to equimolar concentrations (0.6 mM) of ALA or its methyl ester (ALA-ME) showed that the mean total TP production rate during 6 h incubation in serum-free medium was 0.13 fmol cell(-1) h(-1) for ALA-exposed cells compared with 0.04 fmol cell(-1) h(-1) for cells exposed to ALA-ME. Fluorescein diacetate uptake and conversion to fluorescein indicated intracellular non-specific esterase activity, implying that ALA-ME conversion to ALA can occur. Cells exposed to ALA-ME exhibited loss of a greater proportion of total tetrapyrroles in the form of extracellular protoporphyrin IX (PPIX; 22.8%) compared with 11.6% in ALA-treated cells with a corresponding reduction in cell-associated PPIX (P < 0.05). A variable initial elevation in haem levels in ALA-ME-treated cells was observed, but did not reach statistically significant levels.


British Journal of Cancer (1997) **75**(10), 1417–1420
© 1997 Cancer Research Campaign

Short communication

### Comparison of δ-aminolaevulinic acid and its methyl ester as an inducer of porphyrin synthesis in cultured cells

**R Washbrook and PA Riley**

Department of Molecular Pathology, UCL Medical School, The Windeyer Building, 46 Cleveland Street, London W1P 6DB, UK

**Summary** This study was carried out to test the hypothesis that induction of intracellular porphyrin synthesis by δ-aminolaevulinic acid (ALA) used to sensitize cells in photodynamic therapy would be more efficient if the ALA was used in an esterified form. Contrary to expectation, the generation of tetrapyrroles (TP) by cultured epithelial cells (CNCM-I-221) exposed to equimolar concentrations (0.6 mM) of ALA or its methyl ester (ALA-ME) showed that the mean total TP production rate during 6 h incubation in serum-free medium was 0.13 fmol cell$^{-1}$ h$^{-1}$ for ALA-exposed cells compared with 0.04 fmol cell$^{-1}$ h$^{-1}$ for cells exposed to ALA-ME. Fluorescein diacetate uptake and conversion to fluorescein indicated intracellular non-specific esterase activity, implying that ALA-ME conversion to ALA can occur. Cells exposed to ALA-ME exhibited loss of a greater proportion of total tetrapyrroles in the form of extracellular protoporphyrin IX (PPIX; 22.8%) compared with 11.6% in ALA-treated cells with a corresponding reduction in cell-associated PPIX ($P < 0.05$). A variable initial elevation in haem levels in ALA-ME-treated cells was observed, but did not reach statistically significant levels.

**Keywords:** aminolaevulinic acid methyl ester; porphyrin; haem; tetrapyrrole; photodynamic therapy; photosensitizing agents

Exogenous δ-aminolaevulinic acid (ALA) is becoming widely used as an inducer of endogenous synthesis of protoporphyrin IX (PPIX), a photosensitizer used in photodynamic therapy (PDT) (Dilkes et al, 1995; Leveckis et al, 1995; Chang et al, 1996; Fromm et al, 1996). One of the limitations of this technique may be the extent to which ALA can enter the cell. Hence, improving the uptake of ALA would be expected to increase the efficiency of PDT.

Although, at present, the uptake pathway of ALA in mammalian cells is not known, there are various non-specific routes by which ALA may be transported (Washbrook et al, 1997). These include uptake by diffusion or transport by specific membrane proteins responsible for either import, export or both. It is possible that ALA uptake may be altered by modifying groups on the ALA molecule. For example, ALA methyl ester (ALA-ME) resembles ALA except that its carboxyl group is methylated and therefore does not carry a negative charge under physiological conditions. Transport proteins, such as permeases, recognize their substrate by the charged groups specific to the molecules, so altering the groups may inhibit their uptake or alternatively result in the modified molecule being recognized by other transporters (Yudilevich and Boyd, 1987). Export of the molecule may be prevented if a transport protein exists that is responsible for regulating levels of ALA inside the cell.

Because the lipid bilayer is relatively impermeable to charged molecules, passive diffusion through the cell membrane is likely to be governed to some extent by the charge on a molecule. If ALA uptake occurs predominantly by diffusion, the absence of charged groups would be advantageous in that the permeability coefficient of the molecule would be higher.

This paper examines the effectiveness of ALA methyl ester in inducing the synthesis of tetrapyrroles, measured as the sum of cell-associated protoporphyrin IX, haem and extracellular PPIX compared with that of ALA in an established line of mammalian epithelial cells.

*Received 11 October 1996*
*Revised 6 December 1996*
*Accepted 9 December 1996*

*Correspondence to:* R Washbrook

### MATERIALS AND METHODS

### Chemicals

δ-Aminolaevulinic acid (ALA), δ-aminolaevulinic acid methyl ester (ALA-ME), protoporphyrin IX (PPIX), haematin and all other chemicals were obtained from Sigma Chemical Co. Ltd, Poole, UK. ALA and ALA-ME were dissolved in distilled water, filter sterilized and stored at –18°C and defrosted immediately before use.

Trixon X-100 was dissolved in phosphate-buffered saline (PBS; Imperial Laboratories (Europe) Ltd, Andover, UK) to give a concentration of 2%.

### Cells and methods

Epithelial cells between passage number 17 and 25 from an established line derived from rat hepatocytes (CNCM-I-221) were cultured in polystyrene flasks (25 cm$^2$ surface area) in minimal essential Eagle medium (MEM) with Earle's salts, 2 mM L-glutamine, buffered with 20 mM Hepes (Imperial Laboratories). The medium was supplemented with 7.5% sodium bicarbonate, 100 U ml$^{-1}$ penicillin, 100 μg ml$^{-1}$ streptomycin and used with or without fetal bovine serum (FBS) (Imperial Laboratories).

The cells were passaged with trypsin and seeded at approximately $5 \times 10^4$ cells ml$^{-1}$ in medium containing serum (10%) and

Table 1 The relative proportion of tetrapyrrole present as cell-associated PPIX, extracellular PPIX and haem in cells exposed to ALA or ALA-ME for 2, 4 and 6 h

| Exposure time (h) | Percentage of total tetrapyrroles | | | | | | | | |
|---|---|---|---|---|---|---|---|---|---|
| | PPIX | | | | | | Haem | s.d. | *P* |
| | Cell associated | s.d. | *P* | Extracellular | s.d. | *P* | | | |
| ALA | | | | | | | | | |
| 2 | 65.3 | 4.1 | | 13.9 | 1.8 | | 20.8 | 8.9 | |
| 4 | 74.5 | 5.4 | | 10.9 | 1.5 | | 14.6 | 4.0 | |
| 6 | 77.2 | 3.8 | | 10.1 | 1.1 | | 12.6 | 2.3 | |
| ALA-ME | | | | | | | | | |
| 2 | 44.4 | 8.6 | 0.0349 | 26.0 | 1.1 | 0.0012 | 29.6 | 15.6 | 0.529 |
| 4 | 52.0 | 6.4 | 0.0181 | 22.0 | 3.6 | 0.0149 | 26.0 | 14.9 | 0.356 |
| 6 | 61.3 | 2.1 | 0.0061 | 18.9 | 4.0 | 0.0387 | 19.8 | 9.0 | 0.335 |

The values are given as percentages of total tetrapyrrole with standard deviation (s.d.). The statistical significance of the paired comparisons for each time point are shown (*P*).

incubated in loosely capped flasks for 42 h at 37°C in a humidified atmosphere of 2% carbon dioxide.

For experiments, the culture medium was replaced with 5 ml of fresh serum-free medium (SFM) containing 1 μM fluorescein diacetate and 0.6 mM ALA or 0.6 mM ALA-ME, and cells were exposed for periods of 0, 2, 4 and 6 h.

Non-fluorescent fluorescein diacetate was added to the medium, and the amount taken up by the cells and converted to fluorescein was determined. This was used to give an estimation of cellular non-specific esterase activity to ascertain the extent to which ALA-ME conversion to ALA could occur once internalized.

The spent medium was retained, the cells washed three times with PBS and drained. An aliquot of 2% Triton X-100 (3 ml) was added to each flask and incubated for 1 h at room temperature to extract cellular contents, while Trixon X-100 (f.c. 2%) was added to the spent medium to dissolve extracellular porphyrins.

Porphyrins in the medium and in the cells were determined fluorometrically as described previously (Washbrook et al, 1997). The 'HemoQuant' test (Schwartz et al, 1983) was used to determine the intracellular haem levels. Cellular protein was determined using the bicinchoninic acid protein determination kit (Sigma) adapted from a method used by Smith et al (1985), and from this the cell number was calculated using a calibration curve as described by Washbrook et al (1997).

Fluorescein in the cell extract was also measured by fluorimetry (emission at 519 nm, excitation at 491 nm) and the amount of fluorescein per cell was calculated from a calibration curve derived using carboxyfluorescein as standard.

All data points were obtained from triplicate estimations and statistical evaluation was by the two-tailed Student's *t*-test.

### RESULTS AND DISCUSSION

### Total tetrapyrrole production in cells exposed to exogenous ALA and ALA-ME

The total tetrapyrrole was calculated as the sum of the extracellular PPIX, cell-associated PPIX and haem produced per cell over 2, 4 and 6 h. As Figure 1 illustrates, the amount of tetrapyrroles generated in cells exposed to ALA for 6 h reached about 0.75 fmol cell$^{-1}$, which is approximately 3.5 times greater than in cells exposed to ALA-ME for the same period. The rates of tetrapyrrole accumulation over 2, 4 and 6 h, respectively, were 0.144, 0.131 and 0.126 fmol cell$^{-1}$ h$^{-1}$ for cultures exposed to ALA and 0.044, 0.04 and 0.036 fmol cell$^{-1}$ h$^{-1}$ for cultures exposed to ALA-ME. These differences are statistically significant ($P < 0.003$). Table 1 shows that in ALA-ME-treated cells the relative amount of PPIX lost from the cells was greater (on average 22.8 ± 4.2% of the total TP synthesized over 6 h) than in ALA-treated cells (on average 11.6 ± 2.1% of the total TP synthesized over 6 h). This might suggest an effect of the methyl ester on the integrity of the cell membrane. However, no signs of cytotoxicity were evident in cells exposed to ALA-ME. Haem synthesis was not inhibited, haem constituting a higher proportion of total TP in ALA-ME-exposed cells (Table 1), although the differences were not statistically significant.

**Figure 1** The total tetrapyrroles produced per cell after exposure to 0.6 mM ALA (▧) and 0.6 mM ALA-ME (■) for 2, 4 and 6 h. The haem cell$^{-1}$ value obtained from cells before ALA or ALA-ME addition has been deducted from each time point to give total tetrapyrroles induced by ALA or ALA-ME addition. Error bars show standard deviations

© Cancer Research Campaign 1997

**Figure 2** Fluorescein content per cell after exposure to 0.6 mM ALA (▧), 0.6 mM ALA-ME (■) for 2, 4 and 6 h (**A**) and in control cells (□) for 1, 2, 3 and 4 h (**B**). Error bars show standard deviations

**Figure 3** The fluorescence spectra of fluorescein (0.12 mM) and PPIX (0.1 μM) in 2% Triton X-100 when excited with 403 nm wavelength light showing minimal overlap. PPIX does not contribute to the fluorescein emission and approximately equimolar fluorescein contributes less than 1.3% to the PPIX emission at 634 nm

### Fluorescein levels

Fluorescein levels in the medium at 0 h were measured to discount spontaneous hydrolytic conversion of fluorescein diacetate to fluorescein. No significant fluorescence was found.

The fluorescein levels (about 40–100 attomol cell$^{-1}$) reached in cells exposed to either ALA or ALA-ME over 2, 4 and 6 h (Figure 2) imply similar levels of intracellular esterase activity. However, in both cases they exceeded the fluorescein level in control cells, which was about 20–30 attomol cell$^{-1}$. This was not caused by spectral interference of PPIX (see Figure 3). The attainment of a steady-state level of intracellular fluorescein may imply that significant metabolic modification or degradation occurs, and the higher levels and fluctuations in intracellular fluorescein observed in cells exposed to ALA and ALA-ME may reflect secondary effects on the degradation pathway, or possibly export.

Our results show that, under the same conditions, ALA-ME is approximately 70% less effective in stimulating tetrapyrrole synthesis than ALA in cultures of CNCM-I-221 cells. The reason for this could be either that ALA-ME is not taken up by cells so readily as ALA or that ALA-ME remains as a methyl ester once in the cell and cannot be used as a substrate for porphyrin synthesis. However, it is unlikely that the methyl ester remains unhydrolysed, as fluorescein diacetate is taken up by the cells and converted to fluorescein, showing that esterases are active.

The results suggest that ALA transport may be regulated by transport proteins rather than occurring by diffusion. ALA-ME, being an uncharged molecule, would be expected to penetrate cells more readily, whereas the effectiveness of ALA-ME as an inducer of tetrapyrrole synthesis was diminished by comparison with ALA. If ALA enters the cell by a transport pathway, it is possible that either an importer fails to recognize ALA-ME or that an exporter is more effective at expelling ALA-ME than ALA.

Peng et al (1996) have reported the comparative uptake and distribution of ALA and three esters with differing alkyl substitution (methyl, ethyl and propyl) when applied to the mouse skin in the form of a cream. Their data show that ALA is more widely distributed in the body than the corresponding esters, suggesting more rapid transcutaneous uptake of the unesterified amino acid. Our data suggest that, in general, ALA-ME is less effective than ALA at inducing the synthesis of protoporphyrin IX.

### ACKNOWLEDGEMENTS

We thank Dr Sandy MacRobert for helpful discussions. We are grateful to Mrs AMJ Latter and Miss C Johnson for expert technical assistance. This work was supported by the Association for International Cancer Research (AICR).

### REFERENCES

Chang SC, MacRobert AJ and Bown SG (1996) Photodynamic therapy on rat urinary bladder with intravesical instillation of δ-aminolevulinic acid: light diffusion and histological changes. *J Urol* **155**: 1749–1753

Dilkes MG, DeJode ML, Gardiner Q, Kenyon GS and McKelvie P (1995) Treatment of head and neck cancer with photodynamic therapy: results after one year. *J Laryngol Otol* **109**: 1072–1076

Fromm D, Kessel D and Webber J (1996) Feasibility of photodynamic therapy using endogenous photosensitization for colon cancer. *Arch Surg* **131**: 667–669

Leveckis J, Brown NJ and Reed MW (1995) The effect of aminolaevulinic acid-induced, protoporphyrin IX-mediated photodynamic therapy on the cremaster muscle microcirculation *in vivo*. *Br J Cancer* **72**: 1113–1119

Peng Q, Moan J, Warloe T, Iani V, Steen H, Bjorseth A and Nesland J (1996) Build up of esterified aminolevulinic acid derivative-induced porphyrin fluorescence in normal mouse skin. *J Photochem Photobiol B Biol* **34**: 95–96

Schwartz S, Dahl J, Ellefson M and Ahlquist D (1983) The 'Hemoquant' test: a specific and quantitative determination of heme (hemoglobin) in feces and other materials. *Clin Chem* **29**: 2061–2067

Smith PK, Krohn RI, Hermanson GT, Mallia AK, Gartner FH, Provenzano MD, Fujimoto EK, Goeke NM, Olson BJ and Klenk DC (1985) Measurement of protein using bicinchonimic acid. *Anal Biochem* **150**: 76–85

Washbrook R, Fukuda H, Batlle A and Riley P (1997) Stimulation of tetrapyrrole synthesis in mammalian epithelial cells in culture by exposure to aminolaevulinic acid. *Br J Cancer* **75**: 381–387

Yudilevich DL and Boyd CAR (eds) (1987) Amino acid transport in animal cells. In *Physiological Society Study Guide*, Vol. 2, Manchester University Press

*© Cancer Research Campaign 1997*